# Editorial: Community series in novel preclinical model, biomarker, treatment and drug delivery to address immune evasion in cancer, volume II

**DOI:** 10.3389/fimmu.2026.1988140

**Published:** 2026-09-22

**Authors:** Chun-Wai Mai, Kamal Dua, Bey-Hing Goh

**Affiliations:** 1 Centre for Cancer and Stem Cell Research, Institute for Research, Development and Innovation (IRDI), IMU University, Kuala Lumpur, Malaysia; 2 NICM Health Research Institute and School of Science, Western Sydney University, Westmead, NSW, Australia; 3 Woolcock Institute of Medical Research, Macquarie University, Sydney, NSW, Australia; 4 Sunway Biofunctional Molecules Discovery Centre, School of Medical and Life Sciences, Sunway University Malaysia, Selangor Darul Ehsan, Bandar Sunway, Malaysia; 5 Faculty of Health, Australian Research Centre in Complementary and Integrative Medicine, University of Technology Sydney, Ultimo, NSW, Australia; 6 Graduate Institute of Cancer Biology and Drug Discovery, College of Medical Science and Technology, Taipei Medical University, Taipei, Taiwan

**Keywords:** biomarker, cancer, diagnosis, immune evasion, treatment

Cancer diagnosis remains challenging, largely because few biomarkers are sufficiently sensitive and specific for early detection. Tumour‐immune crosstalk adds further complexity, making consistent early‐stage detection difficult (1–3). Without dependable biomarkers to guide clinical decisions, treatment outcomes can be inconsistent (4). Building on our previous editorial (5), we have learned much about immune‐cell‐driven anti‐tumour immunity and how tumours evade these immune responses, alongside promising biomarkers and treatment strategies that target these pathways. In this volume, 16 research groups contribute new work advancing this complex field.

Progress in understanding cancer immune evasion is often held back by the limitations of existing laboratory model systems. Yang et al. addressed this gap by building an integrated preclinical platform for bladder cancer, combining genetically engineered mouse models (GEMMs), single‐cell RNA sequencing and organoid technology. This work delivers scalable, clinically relevant tools that can be used both to unpack immune‐evasion mechanisms and test how well candidate drugs perform. Using another barcoded *KrasG12D* and *Trp53*‐deficient mouse lung adenocarcinoma organoids from the same group (Chen et al.), we also see how these combined approaches can drive biomarker discovery. Collectively, these findings highlight how GEMMs, organoid culture and single‐cell sequencing together can reshape how we study tumour immune escape.

Many manuscripts in this collection report new cancer biomarkers ranging from molecular signatures, microbial profiling to new blood‐derived indices. From multiple myeloma, Tan et al. identified protein‐tyrosine‐phosphatase receptor gamma (PTPRG) as an important mediator of drug resistance and poor patient prognosis. Using single‐cell transcriptomics and functional *in‐vitro* experiments, the group showed that the malignant plasma‐cell sub‐cluster MalPlasma3, marked by high PTPRG expression, displays stem‐like properties and high proliferative potential. This nominates PTPRG both as a prognostic biomarker and a potential therapeutic target for multiple myeloma. For pancreatic ductal adenocarcinoma (PDAC) Wang et al. observed clear differences in biliary microbiota between patients and control participants. The *Muribaculum* strongly correlated with phosphatidic acid (10:0/a‐17:0) and raised CA19‐9 levels, a standard clinical tumour marker for PDAC. Their work suggests profiling biliary microbiota could offer a new predictive biomarker for this disease.

Looking at bladder cancer, Yang et al. used combined bulk and single‐cell RNA‐sequencing data to pinpoint succinylation‐associated genes, namely *KCTD16*, *CD3D*, and *GSDMB*, and described their roles in bladder‐cancer immune evasion. In melanoma, Fan et al. leveraged machine learning together with single‐cell and bulk RNA‐sequencing datasets to generate a Treg‐based gene signature (Treg.Sig) that can predict how patients may respond to immune‐checkpoint inhibitors. Meanwhile, Wang et al. proposed adoptively transferred innate lymphoid cells type 2 innate lymphoid cells (ILC2s)could identify glioblastoma *in vivo*, indicating the possible role of ILC2 in glioblastoma, the most aggressive primary brain tumours.

Moving to readily available clinical blood markers, Shi et al. found that higher pre‐treatment neutrophil‐to‐lymphocyte ratio (NLR) and platelet‐to‐lymphocyte ratio (PLR) were associated with. advanced‐stage neuroblastoma. A combined NLR‐PLR score performed better for risk stratification than either marker alone, though large‐scale phase III trials are still needed for formal clinical validation.

Beyond biomarker identification, several studies also shed light on underlying tumour‐microenvironment mechanisms that drive immune escape. Wu et al. described how increased extracellular‐matrix (ECM) stiffness creates an immunosuppressive tumour niche. High ECM stiffness impairs T‐cell and natural‐killer‐cell activity, favours Treg and M2‐macrophage differentiation, and supports YAP/TAZ‐driven expansion of plasmacytoid dendritic cells. These effects are further amplified when cancer‐associated fibroblasts become activated and trigger epithelial‐mesenchymal transition (EMT). In similar context, Chen et al. studyshowed that epigenetic dysregulation fuels nasopharyngeal carcinoma progression through EMT signalling, further disturbs the tumour microenvironment, enables immune evasion and contributes to treatment failure.

This special issue also features a broad range of preclinical findings, clinical observations, meta‐analyses and reviews focused on new therapeutic approaches and improved drug delivery. Starting with pre‐clinical target work for papillary thyroid carcinoma (PTC), Lv et al. identified plectin (PLEC) as a promising therapeutic target. Few actionable vulnerabilities are known for PTC, but the team validated PLEC function both *in‐vitro* and *in‐vivo*, and provided evidence that troglitazone may exert anti‐tumour activity against PTC via PLEC‐related pathways. For colorectal cancer, Qiu et al. explored cell‐membrane‐coated nanoparticles as a drug‐delivery platform. Combining these nanoparticles with conventional chemotherapy or targeted agents may improve tumour specificity and overall treatment efficacy. In addition, Wang et al. carried out a meta‐analysis covering 26 studies and reported improved clinical outcomes for patients with advanced solid tumours treated with the tyrosine‐kinase inhibitor anlotinib together with PD‐L1 immune‐checkpoint inhibitors. Meanwhile, He et al. reviewed ongoing debates around managing human papillomavirus (HPV)‐associated oropharyngeal carcinoma. They argue that modern treatment planning should integrate immunological and genomic data to build personalised regimens, with the goal of maximising patient survival while minimising avoidable treatment toxicity.

A descriptive case report by Xu et al. offered encouraging real‐world clinical observations. A patient with decompensated hepatitis‐B‐related cirrhosis, advanced hepatocellular carcinoma and lung metastases achieved complete remission after three drug-eluting bead transarterial chemoembolization (DEB‐TACE) sessions, followed by 14 weeks of lenvatinib and 5 months of camrelizumab. No tumour recurrence was seen over 10 months of follow-up. Even so, larger formal studies are required to confirm these promising results. Meanwhile, in the vaccine space, Gao et al. analysed 442 clinical trials investigating melanoma vaccines. While combining melanoma vaccines with immune‐checkpoint inhibitors holds clear therapeutic promise, many candidate approaches fail to progress to phase III trials, leaving high‐level clinical evidence limited.

Taken together, the work assembled in this Research Topic highlights how advances in pre‐clinical platforms, biomarker discovery, and therapeutic development work hand‐in‐hand to unpack cancer immune evasion. Improved model systems such as genetically engineered mouse models paired with organoid and single‐cell sequencing technologies not only help us trace the mechanisms behind tumour immune escape but also serve as critical test‐beds for identifying and validating new candidate biomarkers. From molecular signatures and microbial markers to readily accessible haematological indices, these biomarkers hold real potential to refine risk stratification, predict treatment response, and guide patient‐specific clinical decisions.

Moving from bench toward bedside, the collection also underscores encouraging therapeutic strategies ranging from novel drug targets and smart nanoparticle‐based delivery systems to combination immunotherapies, paving new path for cancer clinics. Nevertheless, many of these promising findings are still rooted in pre‐clinical observations, small‐scale studies or retrospective analyses. Several candidate approaches have yet to complete large‐scale phase III evaluation, creating a well‐recognised translational gap between laboratory discovery and routine patient care.

To bridge this gap, coordinated, multi**-**disciplinary and multi**-**centre trials, supported by thoughtful policy and optimised clinical study design, will be essential. As a collective, these 16 contributions reinforce that no single solution can overcome cancer immune evasion. Instead, sustained progress depends on integrating better pre**-**clinical tools, robust biomarkers, innovative treatment and drug**-**delivery approaches. We hope the findings presented in this volume will stimulate further research, spark cross**-**group collaboration, and ultimately bring tangible clinical benefit for patients facing immune**-**evading cancers.
